# Characterization of Soft Contact Lens Edge Fitting during Daily Wear Using Ultrahigh-Resolution Optical Coherence Tomography

**DOI:** 10.1155/2018/3463595

**Published:** 2018-05-13

**Authors:** Lele Cui, Sisi Chen, Weihe Zhou, Kaixuan Sheng, Lei Zhang, Meixiao Shen, Ming Li

**Affiliations:** ^1^Eye Hospital and School of Ophthalmology and Optometry, Wenzhou Medical University, Wenzhou, Zhejiang, China; ^2^Ningbo Aier Guangming Eye Hosptial, Ningbo, Zhejiang, China

## Abstract

**Purpose:**

To determine conjunctival overlap over the edge of soft contact lens and to visualize the peripheral postlens tear film (PoLTF) underneath soft contact lenses using ultrahigh-resolution optical coherence tomography (UHR-OCT).

**Methods:**

Twenty participants (4 males and 16 females, 23.0 ± 3.7 years) were fitted with two different types of soft contact lenses randomly. The limbus with lens was imaged with the UHR-OCT at the horizontal meridian every two hours up to 6 hours during lens wear. The conjunctival overlap was ranked as the percentage of the edge covered by the conjunctiva. The frequency of occurrence for visualized peripheral PoLTF was determined.

**Results:**

The average conjunctival overlaps at insertion were 49% and 73% for galyfilcon A and balafilcon A lenses and increased significantly to 84% and 90% by 6 hours of lens wear (*P* < 0.001). Lenses with rounded edges had more conjunctival overlap than the lenses with angled edges (*P*=0.014). There were significant decreases for PoLTF on the conjunctiva (*P*=0.014) and peripheral cornea (*P*=0.004) over the study period compared to insertion. The percentage of subjects with PoLTF on the conjunctiva (32.5%) and peripheral cornea (36%) were greater in subjects wearing balafilcon A lenses (*P*=0.017).

**Conclusions:**

Increased conjunctival overlap over the lens edges and reduced PoLTF underneath the peripheral region of soft contact lenses were shown during lens daily wear. The lens edge configuration may play a role in conjunctival response and peripheral PoLTF.

## 1. Introduction

When a soft contact lens, between 14 and 14.5 cm in diameter, is worn on the eye, it completely covers the cornea and overlaps approximately 2 mm on to the bulbar conjunctiva [1]. Additionally, in the course of eye movements and blinking, the lens may momentarily become displaced and overlap further onto the bulbar conjunctiva, perhaps up to 4-5 mm from the limbus. The interactions between contact lens and conjunctiva, such as the encroachment onto the lens edge, have been reported associated with conjunctival indentation and conjunctival flaps [2]. A poor-fitting contact lens may induce clinical complications of the conjunctiva, including physical irritation that results excess staining [3], deep arcuate band staining caused by pressure from the lens edge, hyperemia, and chemosis [4]. So wearing contact lens has the influence not only on the physiology of the cornea but also on the conjunctiva. The postlens tear film (PoLTF) plays important roles in contact lens fitting. It cushions and lubricates the lens movement on the ocular surface [5–7] and provides oxygen transmission to the cornea [8]. The depletion of the PoLTF may cause lens adherence [9] and ocular surface staining [10], all of which are characteristic of contact lens-associated dry eye [11]. These complications may result in contact lens discontinuation [12]. Measurements of the central PoLTF have been the subject of several previous studies [13–15]. The central PoLTF is several micrometers in thickness, and PoLTFs decrease further after lens insertion [14]. However, not much is known about the PoLTF on the peripheral cornea and conjunctiva during lens wear.

With the advent of ultrahigh-resolution optical coherence tomography (UHR-OCT), it is possible to image the contact lens edge at the conjunctiva and visualize the PoLTF underneath the periphery of the lens [11, 16]. Evaluating the diurnal variation of conjunctival responses related to the contact lens and peripheral PoLTF may provide insightful information for a better understanding of the change of the lens edge fitting. The goal of this study was to measure the conjunctival overlap over the lens edge and determine the PoLTF underneath the peripheral region of soft contact lenses during daily wear by UHR-OCT.

## 2. Subjects and Methods

This study was approved by the Office of Research Ethics of the Wenzhou Medical University and was conducted in accordance with the tenets of the Declaration of Helsinki. Informed consent was obtained from each participant prior to enrollment in the study. Twenty healthy subjects (4 males and 16 females; mean ± standard deviation age, 23.0 ± 3.7 years) with no previously diagnosed dry eye and with no dry eye symptoms or ocular surface disease were recruited for the study.

To observe the lens edges and interaction with the ocular surface and detect the presence of the peripheral PoLTF, a custom built, high speed, UHR-OCT instrument with 3 *μ*m resolution was used for this study [11, 16]. Briefly, the light source was a three-module superluminescent diode (Broadlighter, T840-HP, Superlum diodes Ltd., Co., Cork, Ireland) with a center wavelength of 840 nm and a full width at half maximum bandwidth of 100 nm. The power of the incident light delivered into the anterior segment was lowered to 750 *µ*W to ensure the safety of the eye.

All subjects were tested in a consulting room with controlled temperature (15–25°C) and humidity (30%–50%) after 10 AM to avoid the edematous cornea and sleep-induced alterations of the tear film [17]. Two eyes of each subject were fitted with two different types of soft contact lenses (Table 1). The order of these two lenses was randomized for each eye of each subject. OCT images were taken immediately after lens insertion and at 2, 4, and 6 hours during lens wear. The subjects were asked to sit in front of the instrument and look straight at an external target while an 8 mm-width scan was made on the horizontal meridian. The limbal images for the temporal side were obtained for each eye by rotating the OCT probe to target the limbus while the subject fixated on the target with the primary gaze.

**Figure 1:**
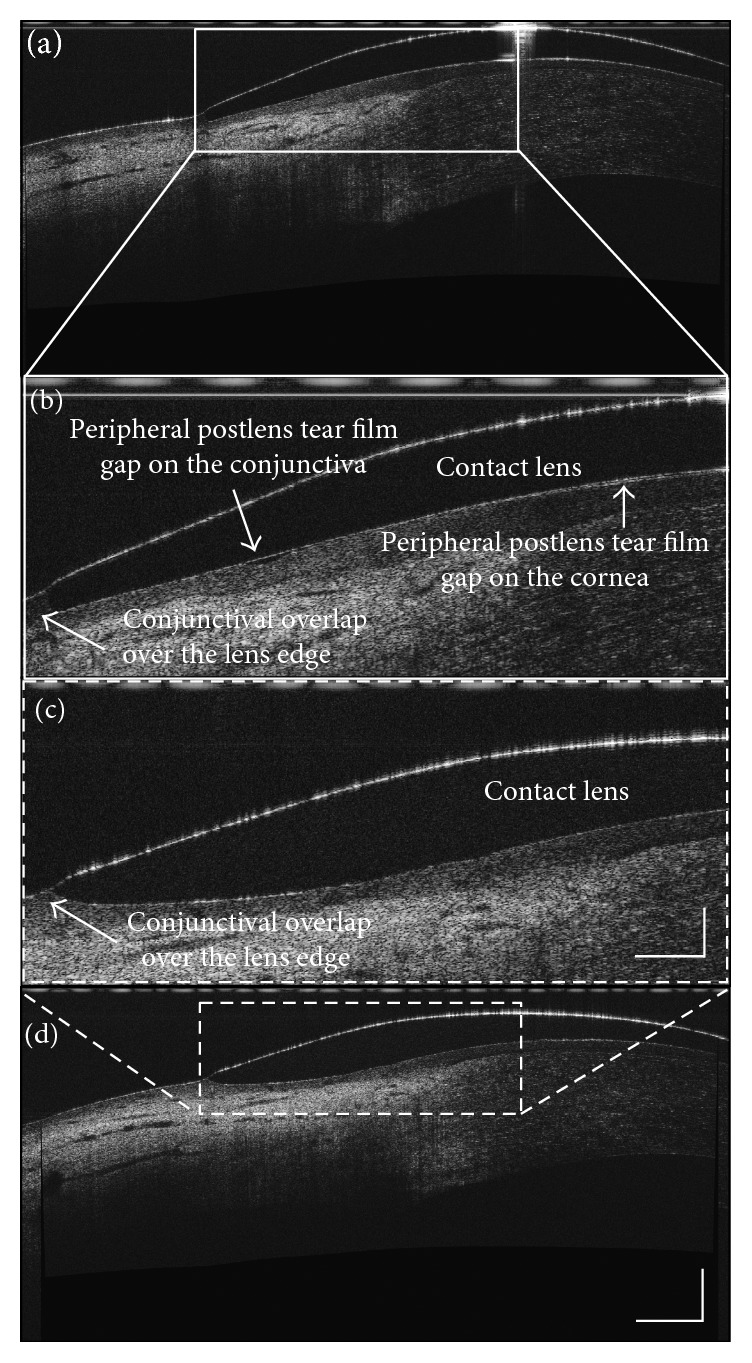
Edge conjunctival coverage and PoLTF underneath the peripheral lens. Conjunctival response to soft contact lens was characterized as an overlap of conjunctival tissue at the edge of the lens. The PoLTFs on the cornea and on the conjunctiva were clearly visualized as gaps between the lens and the ocular surfaces in (b). One was located at the peripheral cornea, and the other was located at the limbal transition to the conjunctiva. (b) and (c) were the magnified images of (a) and (d). (a) and (b) are balafilcon A lenses. (c) and (d) are galyfilcon A lenses. The bars denote 100 *µ*m for two of the images (a) and (d) and 250 *µ*m for the other two images (b) and (c).

Because UHR-OCT images of the contact lens edges were optically distorted due to the different refractive indices and curved surfaces [18, 19] custom software was used to correct the image using Fermat's principle [18]. The percentage of edge covered by the conjunctiva was categorized by an analog ranking scale of 0%, 25%, 50%, 75%, and 100% for each lens after correction for the optical distortion [17]. Images labeled as 0% edge coverage showed almost no conjunctival overlap over the temporal lens edges. Images labeled as 100% edge coverage had conjunctival overlap that covered almost the entire temporal lens edges (Figures 1 and 1). In the OCT images, the peripheral PoLTF was visualized as a gap between the corneal or limbal surfaces and the posterior surface of the lens (Figure 1). The gaps on the cornea and conjunctiva were visualized and ranked. Each image was inspected and ranked as “1” if a gap was presented on the cornea and the conjunctiva. It was ranked as “0” as the gap was absent [17]. The observer (ML) was masked to the lens types to minimize bias during evaluation of the edge coverage and PoLTF gaps.

Linear mixed model for edge ranking and generalized estimating equation (GEE) for gaps on the conjunctiva and cornea was used to estimate the contact lenses group difference and over time change of the variables. Least significant difference (LSD) was performed for post hoc comparisons between any two time points. The Wilcoxon Rank-Sum test and the chi square test were utilized for intergroup comparisons at each time point. Data analysis was performed using IBM SPSS Statistics (Version 20.0, IBM Corp., Armonk, NY, USA). *P* < 0.05 was considered significant.

## 3. Results

**Figure 2:**
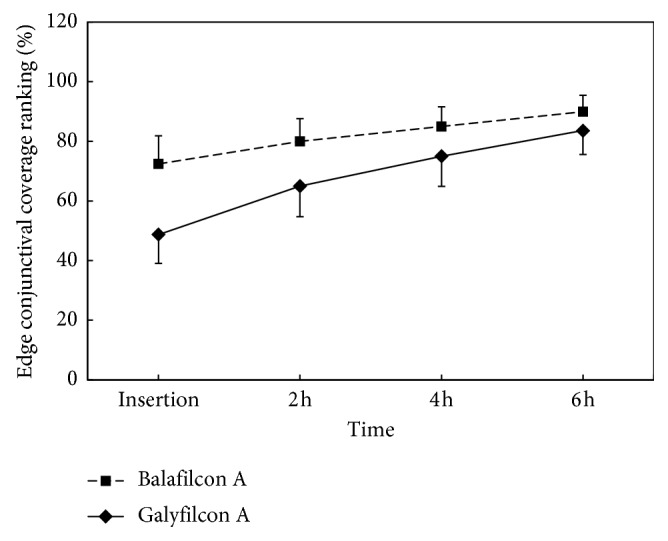
Time-dependent overlap of conjunctiva at lens edge. The average conjunctival overlap increased significantly at 6 hours after insertion. Balafilcon A lenses had more conjunctival overlap than the galyfilcon A lenses. Galyfilcon A lenses had showed greater increase in tendency of conjunctival overlap over time. Bars denote 95% confidence interval (CI).

The average conjunctival overlaps at insertion were 49% and 73% for galyfilcon A and balafilcon A lenses, respectively. The values were increased significantly by 6 hours of lens wear (*F*=27.60, *P* < 0.001; Table 2), which reached 84% and 90%. Lenses with rounded edges (balafilcon A lenses) had more conjunctival overlap than the galyfilcon A lenses with angled edges (*F*=6.58, *P*=0.014; Table 2). Galyfilcon A lenses had showed greater increase tendency of conjunctival overlap over time (*F*=3.08, *P*=0.039; Figure 2 and Table 2).

**Figure 3:**
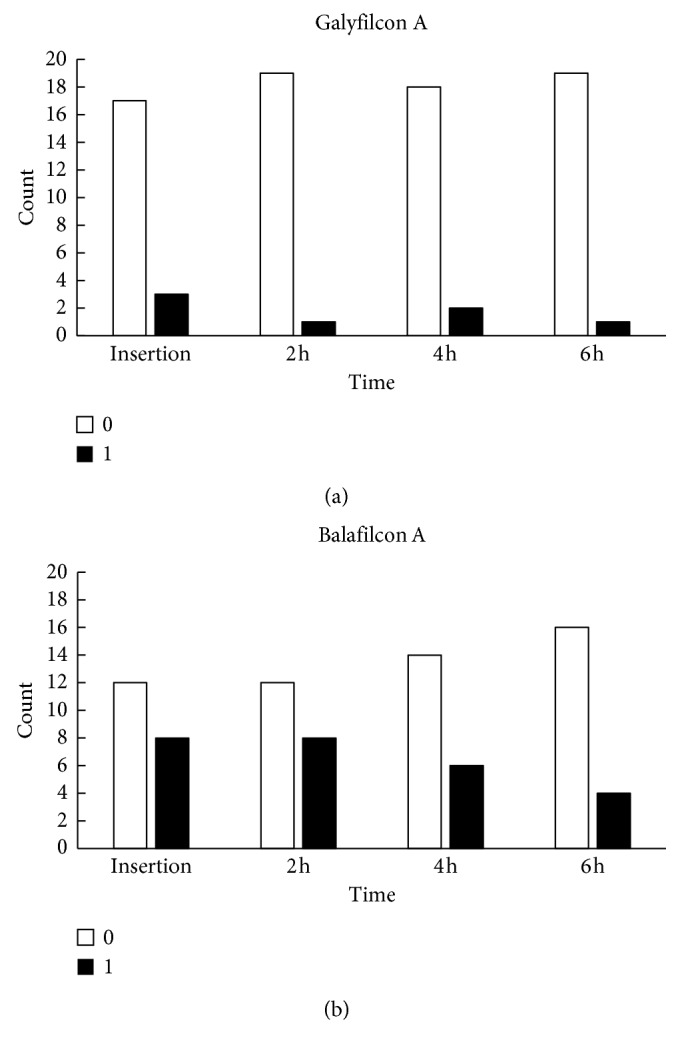
Time-dependent changes in PoLTF at the limbus. It was ranked as “1” if a gap was presented on the conjunctiva and “0” as the gap was absent. (a) Time-dependent changes in PoLTF at the limbus for galyfilcon A lenses and (b) balafilcon A lenses. Frequency of occurrence for PoLTF on the conjunctiva decreased over the study period compared to insertion.

**Figure 4:**
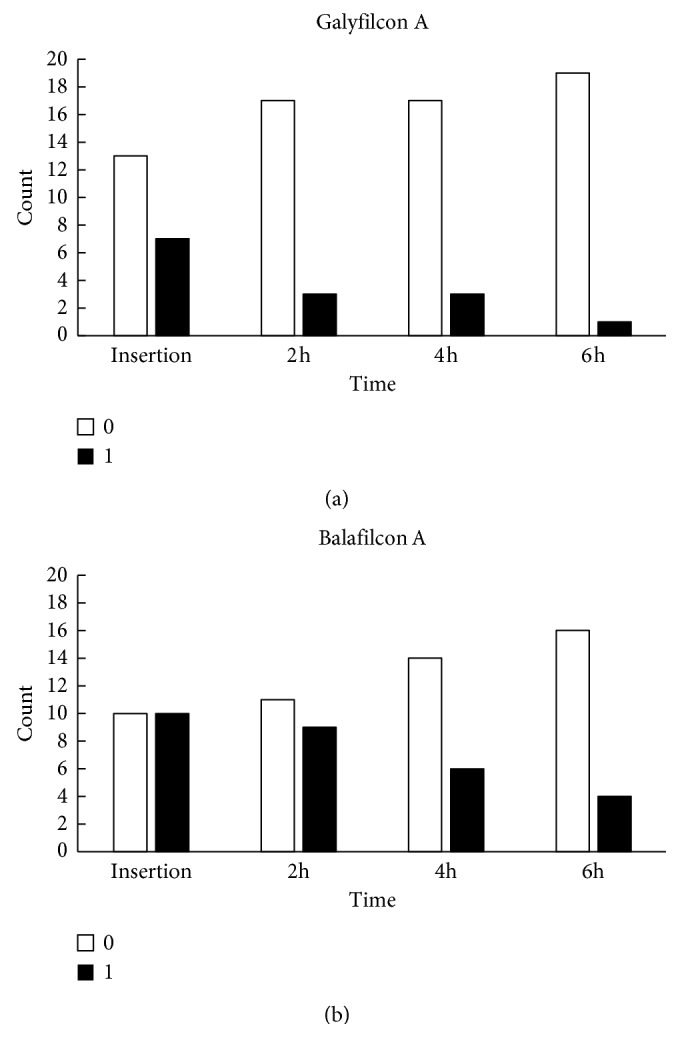
Time-dependent changes in PoLTF on the cornea. It was ranked as “1” if a gap was presented on the cornea and “0” as the gap was absent. (a) Time-dependent changes in PoLTF on the cornea for galyfilcon A lenses and (b) balafilcon A lenses. Frequency of occurrence for PoLTF on the cornea decreased over the study period compared to insertion.

Limbal PoLTF was visualized at 15% at insertion and 5% at 6 hours for galyfilcon A lenses, and the value went from 40% to 20% for balafilcon A lenses (Wald *χ*^2^=10.58, *P*=0.014; Figure 3 and Table 2). The percentage of subjects with PoLTF around the limbus was greater in subjects wearing balafilcon A (32.5%) compared to galyfilcon A lenses (7.5%) (Wald *χ*^2^=5.70, *P*=0.017; Figure 3 and Table 2). There were significant decreases for PoLTF on the peripheral cornea over the study period compared to insertion (Wald *χ*^2^=13.08, *P*=0.004; Figure 4 and Table 2). For 35% of eyes, the PoLTF was visualized at the peripheral cornea at insertion and in 5% after 6 hours of lens wear for galyfilcon A lenses. The PoLTF was visualized at the peripheral cornea at insertion in 50% of eyes and in 20% after 6 hours of lens wear for balafilcon A lenses. More subjects, 36% in average, wearing the balafilcon A lenses had PoLTF present at the peripheral cornea than those wearing galyfilcon A lenses (Wald *χ*^2^=4.18, *P*=0.041; Figure 4 and Table 2).

## 4. Discussion

Evaluating the interactions between soft contact lenses and the ocular surface, especially at the interface with the limbus and conjunctiva, has been a challenge because of the anatomical characteristics of this tissue and the limitation of imaging techniques. Few studies have produced images of the interaction between the soft contact lens and conjunctiva due to the relatively low resolution of the OCT image. With the rapid development of OCT technology, high- or ultrahigh- resolution OCT images can now be obtained in contact lens practices [20, 21]. We previously demonstrated different contact lens edge configurations and the presence of the peripheral PoLTF by UHR-OCT [11, 16]. In the present study, we aimed at evaluating the conjunctival response to soft contact lens which was characterized as an overlap of conjunctival tissue at the edge of the lens and the presence of the peripheral PoLTF during the daily wear.

The conjunctival overlap was evident and different between two soft contact lenses investigated in this study. The difference may be because of different pressure profiles [22] produced across the ocular surface underneath each lens [23]. When a lens is fitted on the eye, it must flex to align with the ocular surface. Using finite element analysis, pressure profiles of soft contact lenses on the eye were simulated, and local pressures were projected to exist around the lens edge and midperiphery of the cornea (Evans SR, et al. IOVS 2005; 46: ARVO E-Abstract 2059; Hofmann G, et al. IOVS 2010; 51: ARVO E-Abstract 3418). Compared to the cornea, the conjunctiva is composed of softer tissue (lower elastic modulus) which means that the conjunctiva may be easy to deform and build up around the lens edge [24]. Lens diameter, lens power, base curvature, and lens thickness profile may influence the level of local pressures [25]. In the present study, the values of central lens thickness, base curvature, diameter, and power for two lenses were very close. Contact lenses with rounded edges produced more conjunctival overlap than angled edges. Our results presented here indicate that edge shape and lens design are likely to affect lens-induced pressure and consequently affect conjunctival overlap.

The conjunctival overlap increased by six hours of lens wear, suggesting that wearing time may be another factor contributing to the overlap. Because tear meniscus volume is reduced after short-term lens wear [26, 27], especially at the end of the day, the lens may become dehydrated. Consequently, the lens dehydration or shrinkage might change the pressure profiles on the ocular surface and increase the lens edge tip pressure, thus resulting in more conjunctival overlap.

In particular, the existence of the PoLTF at the corneal periphery or conjunctiva likely indicates the presence of higher localized pressure points. Two touch points may create a pocket or gap that contains the PoLTF. In the present study, the PoLTF at the peripheral cornea and at the limbal transition zone were clearly visualized in a portion of the subjects for up to 6 hours. Subjects wearing balafilcon A lenses with rounded edges were more likely to have a peripheral PoLTF than subjects wearing the galyfilcon A lenses with angled edged, which was similar to our previous results [16]. Besides, the shape of ocular surface affects the fit of a lens, and significant differences in the peripheral PoLTF between two different lenses also indicate that lens designs play important roles. Our results here may indicate that round edged contact lenses have higher localized pressure near the lens edge and at the midperiphery of the cornea. This could result in the persistence of the PoLTF. Lenses with a high modulus were found to have more movement [28]. More movement in a lens with a high modulus might be attributed to the difficulty of deformation and the adherence to the ocular surface that may result in a high frequency of occurrence for peripheral PoLTF.

Lens wearing time may be another factor contributing to the changes of the peripheral PoLTF. At the peripheral cornea as well as limbus, the number of subjects in which the PoLTF could be visualized decreased during the 6 h of lens wear. Over a period of time, lenses appear to deform and conform to the ocular surface [29], and lid tension during blinking may facilitate the deformation of the lens, both of which may explain the diminished peripheral PoLTF on the cornea and limbus.

There were some limitations in the present study. We did not take into account lens movement that may play a role in the conjunctival overlap. The PoLTF at the periphery was visualized but not quantified with respect to size and location. We only evaluated the conjunctival overlap and PoLTF at the horizontal meridian. As this was the first attempt to characterize the edge fitting properties of soft contact lenses during daily wear, the role of these variables will be considered in future studies. Linking the shape of the ocular surface and the lens edge fitting and three-dimensional quantitation of the size and location of the peripheral PoLTF may be necessary to fully understand the overall lens edge fitting.

In summary, evaluation by UHR-OCT of soft contact lens wear over a 6-hour period showed increased conjunctival overlap over the lens edges and reduced PoLTF underneath the peripheral region of soft contact lenses. The lens edge configuration may play a role in conjunctival response and peripheral PoLTF. UHR-OCT is well suitable for evaluating the lens edge fitting during daily soft contact lens wear.

## Figures and Tables

**Table 1:** Design parameters of contact lenses measured in the study.

	PureVision	Acuvue Advance
Manufacturer	Bausch & Lomb	Vistakon, Johnson & Johnson
Diameter (mm)	14.0	14.0
Base curvature (mm)	8.6	8.7
Power (D)	−3.00 D	−3.00 D
Material	Balafilcon A	Galyfilcon A
Modulus (MPa)	1.1	0.43
Edge shape	Rounded	Angled
Center thickness (mm)	0.09	0.07
Water content (%)	36	47

**Table 2:** Conjunctival response and peripheral PoLTF for two lenses during lens daily wear.

Lens	Edge conjunctival coverage ranking (%)			Peripheral postlens tear film at the limbus			Peripheral postlens tear film on the cornea		
	$\bar{x}\pm s$	*F*	*P*	*n* (%)	Wald *χ*^2^	*P*	*n* (%)	Wald *χ*^2^	*P*
A	68.12 ± 25.15	23.18	<0.001	7 (8.75%)	786.47	<0.001	14 (17.5%)	9.35	0.025
B	81.88 ± 17.78	8.75	0.001	26 (32.50%)	2.87	0.413	29 (36.25%)	6.49	0.09
A + B	75.00 ± 22.78	27.60^a∗^	<0.001^a^^∗^	33 (20.63%)	10.58^a^^∗^	0.014^a^^∗^	43 (26.88%)	13.08^a^^∗^	0.004^a^^∗^
Between A and B	(*F*=6.58, *P*=0.014)^b^^∗^			(*χ*^2^=5.70, *P*=0.017)^b^^∗^			(*χ*^2^=4.18, *P*=0.041)^b^^∗^		
	(*F*=3.08, *P*=0.039)^#^			(*χ*^2^=6.79, *P*=0.079)^#^			(*χ*^2^=2.62, *P*=0.454)^#^		

Lens A: galyfilcon A; lens B: balafilcon A; ^∗^*F* statistic and *P* value of the main effect (lens and time); ^#^*F* statistic and *P* value of interaction; ^a^differences over time; ^b^differences between lenses.

## Data Availability

The data used to support the findings of this study are available from the corresponding author upon request.
